# Alpha-Glucosidase Promotes Hemozoin Formation in a Blood-Sucking Bug: An Evolutionary History

**DOI:** 10.1371/journal.pone.0006966

**Published:** 2009-09-09

**Authors:** Flávia Borges Mury, José Roberto da Silva, Ligia Souza Ferreira, Beatriz dos Santos Ferreira, Gonçalo Apolinário de Souza-Filho, Jayme Augusto de Souza-Neto, Paulo Eduardo Martins Ribolla, Carlos Peres Silva, Viviane Veiga do Nascimento, Olga Lima Tavares Machado, Marília Amorim Berbert-Molina, Marilvia Dansa-Petretski

**Affiliations:** 1 Laboratório de Química e Função de Proteínas e Peptídeos, Universidade Estadual do Norte Fluminense, Campos dos Goytacazes, Rio de Janeiro, Brazil; 2 Laboratório de Biotecnologia, Universidade Estadual do Norte Fluminense, Campos dos Goytacazes, Rio de Janeiro, Brazil; 3 Instituto de Química, Departamento de Bioquímica and NUPEM, Universidade Federal do Rio de Janeiro, Macaé, Rio de Janeiro, Brazil; 4 Instituto Nacional de Ciência e Tecnologia em Entomologia Molecular (INCT-EM), Rio de Janeiro, Rio de Janeiro, Brazil; 5 Departamento de Parasitologia, Universidade Estadual de São Paulo, Botucatu, São Paulo, Brazil; 6 Departamento de Bioquímica, Universidade Federal de Santa Catarina, Florianópolis, Santa Catarina, Brazil; 7 Department of Molecular Microbiology and Immunology, Johns Hopkins University, Bloomberg School of Public Health, Baltimore, Maryland, United States of America; Universidade Federal do Rio de Janeiro (UFRJ), Instituto de Biofísica da UFRJ, Brazil

## Abstract

**Background:**

Hematophagous insects digest large amounts of host hemoglobin and release heme inside their guts. In *Rhodnius prolixus*, hemoglobin-derived heme is detoxified by biomineralization, forming hemozoin (Hz). Recently, the involvement of the *R. prolixus* perimicrovillar membranes in Hz formation was demonstrated.

**Methodology/Principal Findings:**

Hz formation activity of an α-glucosidase was investigated. Hz formation was inhibited by specific α-glucosidase inhibitors. Moreover, Hz formation was sensitive to inhibition by Diethypyrocarbonate, suggesting a critical role of histidine residues in enzyme activity. Additionally, a polyclonal antibody raised against a phytophagous insect α-glucosidase was able to inhibit Hz formation. The α-glucosidase inhibitors have had no effects when used 10 h after the start of reaction, suggesting that α-glucosidase should act in the nucleation step of Hz formation. Hz formation was seen to be dependent on the substrate-binding site of enzyme, in a way that maltose, an enzyme substrate, blocks such activity. dsRNA, constructed using the sequence of α-glucosidase gene, was injected into *R. prolixus* females' hemocoel. Gene silencing was accomplished by reduction of both α-glucosidase and Hz formation activities. Insects were fed on plasma or hemin-enriched plasma and gene expression and activity of α-glucosidase were higher in the plasma plus hemin-fed insects. The deduced amino acid sequence of α-glucosidase shows a high similarity to the insect α-glucosidases, with critical histidine and aspartic residues conserved among the enzymes.

**Conclusions/Significance:**

Herein the Hz formation is shown to be associated to an α-glucosidase, the biochemical marker from Hemipteran perimicrovillar membranes. Usually, these enzymes catalyze the hydrolysis of glycosidic bond. The results strongly suggest that α-glucosidase is responsible for Hz nucleation in the *R. prolixus* midgut, indicating that the plasticity of this enzyme may play an important role in conferring fitness to hemipteran hematophagy, for instance.

## Introduction

During the course of evolution, hematophagous organisms have developed and selected an array of strategies to counteract heme cytotoxicity to adapt successfully to blood feeding [1]. Since the midgut of hematophagous insects is the first site that comes into contact with huge amounts of heme (iron-protoporphyrin IX) during hemoglobin digestion, it is not surprising to encounter efficient manners of reducing heme availability in this organ [2]–[4]. Graça-Souza [5] reviewed some important adaptations of hematophagous organisms to circumvent heme toxicity; some of these include heme-binding proteins, antioxidant enzymes, low molecular mass antioxidants, heme degradation and Hz formation.

*R. prolixus* is a hematophagous hemipteran that sequesters hemoglobin-derived heme into a dark-brown pigment named Hz which is an insoluble and less reactive substance [2]. This is the first line of defense against heme toxicity in the midgut of this insect [6]. Hz was first described in *Plasmodium falciparum* [7], but over the last decade Hz has been described in a number of other blood-sucking organisms such as the helminth worms, *Schistosoma mansoni* [8] and *Echinostoma trivalvis* [9], and the bird-infecting protozoan, *Hemoproteus columbae* [10]. These discoveries have broadened the interest into understanding the mechanism of Hz formation.

Despite the descriptions of Hz in some different organisms, there is a great discussion about the process of Hz formation. Some evidence suggests lipids to be catalysts in this process by increasing the heme solubility in acidic conditions [11]. Lipids has gained more interest, due to the fact that some authors demonstrated the presence of neutral lipid bodies closely associated with the digestive vacuole of *P. falciparum* [12]. Other authors went on to show that Hz is entirely encapsulated within lipid bodies both in *P. falciparum* [13] and *S. mansoni* [14], suggesting that Hz formation is achieved in a lipid environment. The role of the water-lipid interface is also discussed in Hz formation, since the Hz is formed at the interface of lipids and aqueous phases, seeming to be essential to the process [15].

Even though lipids present a very important role in Hz formation processes, the participation of proteins in this process has also been proposed [16]. These authors showed that histidine-rich protein-II (HRP-II) is capable of promoting Hz formation by a process mediated by histidine residues at the active site. HRP-II might facilitate Hz formation by binding with a large number of heme molecules, and facilitating dimer formation involving iron-carboxylate bonds between every two heme molecules [17]. However, parasites lacking HRP are still capable to promote Hz formation [18]; as such the role of proteins as a nucleation site for Hz growth should not be discarded. Recently, a novel *Plasmodium* Heme Detoxification Protein (HDP) has been reported to induce the conversion of heme into Hz [19].

Perimicrovillar membranes (PMM) are extracellular structures present in Hemipteran and Tysanopteran insects and α-glucosidase was shown to be an enzyme marker of these PMM in *R. prolixus* and *Dysdercus peruvianus* midgut cells [20]. This enzyme has only one subunit and catalyses the hydrolysis of different ingested substrates (sucrose, maltotriose, maltotetraose, soluble starch and ρ-nitrophenyl *α*-D-glucopyranoside) at the same active site, which seems to have five subsites [21].

The PMM promote Hz formation and we have previously demonstrated that the protein moiety of the PMM is involved in this process [22]. Here, we show that α-glucosidase from *R. prolixus* midgut promotes Hz formation, both *in vivo* and *in vitro*. To our knowledge, this is the first report of the involvement of a carbohydrase in this heme detoxification process.

## Results

### Effect of α-glucosidase inhibitors on Hz formation

**Figure 1:**
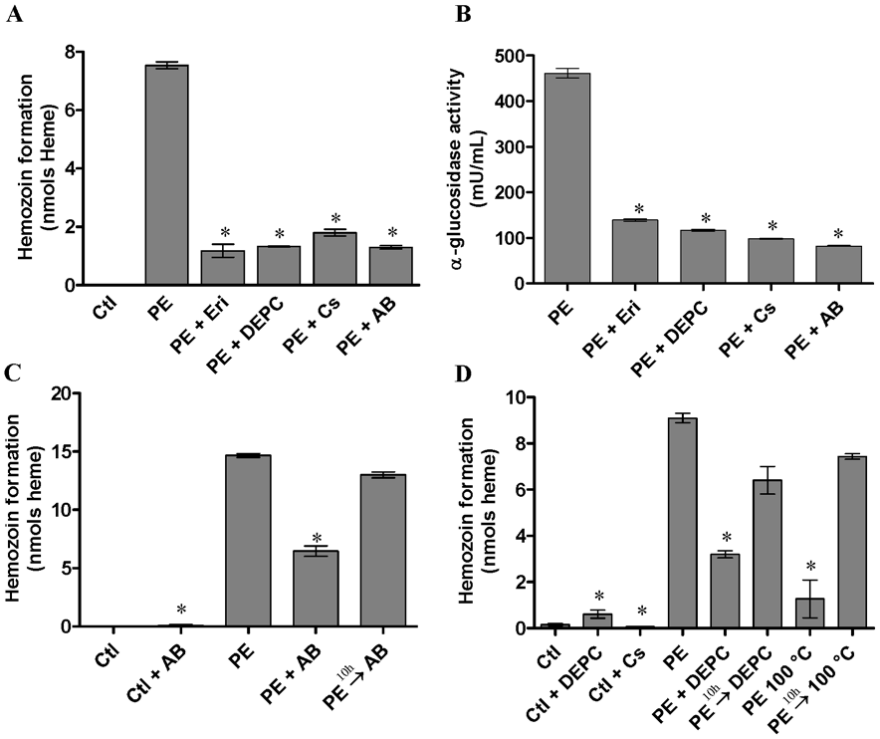
Hz formation and α-glucosidase activities in the presence or absence of inhibitors *in vitro*. A. C. and D. Hz formation. B. α-glucosidase activity. Ctl - hemin; PE - protein extract of midgut epithelium; PE + Eri - protein extract + erythritol; PE + DEPC - protein extract + diethypyrocarbonate; PE + Cs - protein extract + castanospermine; PE + AB - protein extract + anti *D. peruvianus* α-glucosidase antibody; Ctl + AB - hemin + antibody; Ctl + Cs - hemin + castanospermine; Ctl + DEPC - hemin + diethypyrocarbonate; PE → AB - protein extract + antibody 10 hours after starting assay; PE → DEPC - protein extract + diethypyrocarbonate 10 hours after starting assay; PE 100°C - protein extract boiled for 10 min before starting assay; PE → 100°C - protein extract boiled 10 hours after starting assay. The assays of Hz formation were carried out for 24 h at 28°C as described in materials and methods. Hz formation activity was expressed as nmol heme aggregated in 24 h for 15 µg protein extract. The assays of α-glucosidase activity were determined using a colorimetric method. Unless otherwise indicated, activity was expressed as nmol *ρ*-nitrofenolate released in 1 min. Results shown are means ±SEM (n = 4) of two experiments run in triplicate. The experiments with inhibitors were significantly different from protein extract alone *(*P*<0.05).

Erythritol (100 mM) and castanospermine (30 µM), specific inhibitors of α-glucosidase, Diethypyrocarbonate (DEPC) (10 mM), which react with and modify histidine residues, and a polyclonal antibody raised against α-glucosidase from *D. peruvianus* (1∶2500) were used in order to investigate the correlation between the α-glucosidase and Hz formation activities (Figure 1A and 1B). Erythritol is a competitive inhibitor of the α-glucosidase assayed with NPαGlu [23]; only one molecule of these inhibitors is able to bind at putative sub-sites 1 or 2 of this enzyme. Here, the effect of erythritol and castanospermine on α-glucosidase activity and Hz formation were tested. The results show that both activities were very sensitive to these agents. DEPC is used to reveal mechanistic differences among α-glucosidases from mammalian, plant and yeast cells [24]–[26]. For the *R. prolixus* α-glucosidase, DEPC strongly inhibited this enzyme as well as the Hz formation, suggesting that histidine residue (s) at or close to the catalytic domain of the enzyme to be important for both activities. An antibody raised against α-glucosidase of the phytophagous hemipteran *D. peruvianus*, which also successfully recognizes the enzyme in the PMM of the *R. prolixus* midgut [20], was able to drastically inhibit Hz formation and α-glucosidase activities *in vitro* (Figure 1A and 1B). The next set of experiments was designed to evaluate whether α-glucosidase acts as a nucleation site for the process of Hz formation. To test this hypothesis, the Hz formation assay was carried out using the midgut protein extract from insects previously fed on blood. Additions of the *D. peruvianus* anti-α-glucosidase antibody (Figure 1C) or DEPC (Figure 1D), 10 hours after the onset of the experiment, were not able to inhibit the hemozoin formation, since it had already been nucleated. As a corroborative datum, a 10 min boiling step of the protein extract preceding the start of Hz formation assay reduced its ability to initiate Hz nucleation. Moreover, boiling the assay mixture 10 hours after the onset of the assay did not interfere in the continuing occurrence of the Hz formation process (Figure 1D). These results suggest that the enzyme is implied in the process of Hz formation, particularly on nucleation step, since specific α-glucosidase inhibitors were able to interfere in the Hz formation.

### Effect of maltose on Hz formation

**Figure 2:**
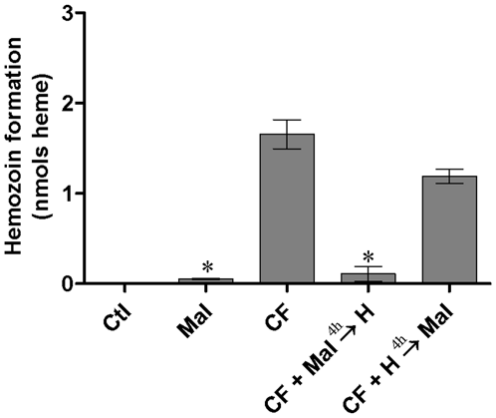
Hz formation activity in the presence or absence of maltose *in vitro*. Ctl - hemin; Mal - Maltose; CF - chromatographic fraction; CF + Mal → H - chromatographic fraction + maltose and hemin added 4 h after starting assay; CF + H → Mal - chromatographic fraction + hemin and maltose added 4 h after starting assay. The assays of Hz formation were carried out for 24 h at 28°C as described in materials and methods. Hz formation activity was expressed as nmol of aggregated heme, during 24 h, for 8 µg protein. The results are the mean and standard deviation of one experiment run in triplicate. The experiment where maltose was added before hemin was significantly different from that with protein alone or that with hemin being added first *(*P*<0.05).

The action of competitive inhibitors of α-glucosidase (DEPC, castanospermine and erythritol) inhibiting the Hz formation suggests that the same site can be involved in both activities. In order to address this question, the Hz formation activity in the presence of both maltose (30 mM), an α-glucosidase substrate, and hemin was tested. Ion exchange chromatographic fraction containing α-glucosidase activity was firstly incubated with maltose for 4 hours. After this time, hemin was added but Hz formation did not occur (Figure 2). Primary incubation of chromatographic fraction with acetate buffer containing hemin, followed by the addition of maltose, did not interfere in the efficiency to sustain Hz formation (Figure 2). The results show that the binding of maltose to the enzyme blocked the Hz formation activity. Since the binding of maltose and hemin are mutually exclusive processes, the simplest explanation is that both share a common binding site.

### RNAi mediated knock-down of α-glucosidase reduces Hz formation

**Figure 3:**
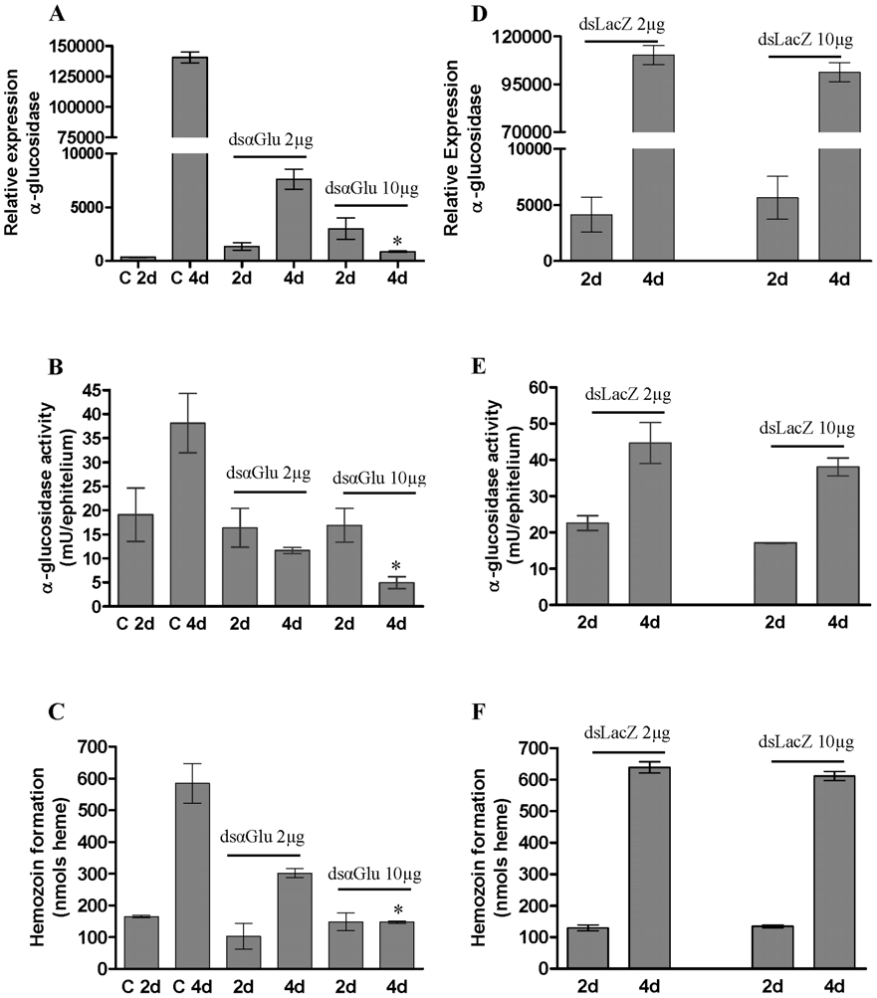
Silencing of α-glucosidase by injection of dsRNA and its action in *R. prolixus* “in vivo”. A: Relative expression (qPCR) of α-glucosidase in the midgut after injection of dsαglu; B: α-glucosidase activity into midgut of insects injected with dsαglu; C: Hz produced by the insects injected with dsαglu; D: Relative expression of α-glucosidase (qPCR) in the midgut after injection of dsLacZ; E: α-glucosidase activity into midgut of insects injected with dsLacZ; F: Hz produced by the insects injected with dsLacZ. The insects were injected with 2 µL of 100 mM PBS pH 7.4 (control), dsLacZ (2 or 10 µg/female) or dsα-glu (2 or 10 µg/female) and analyzed 2 or 4 days after feeding on blood. Hz measurement was carried out using a pool of six midgut epithelium. Four replicates were performed. Each replicate consists of a pool of six adult females. The insects injected with 10 µg dsRNA after 4 days of feeding were significantly different from control 4 day insects *(*P*<0.05).

The effects of double-stranded α-glucosidase *Anopheles aquasalis* (dsαGlu) injection on α-glucosidase activity and Hz formation were evaluated in order to correlate both activities in the *R. prolixus* midgut. Then two experiments were performed. In experiment 1 degenerated primers, based on the sequence of *Anopheles aquasalis* α-glucosidase gene were used as templates for dsαGlu synthesis. Injections of dsαGlu caused a reduction in gene expression, as revealed by qPCR analysis (Figure 3A). This effect was more pronounced with 10 µg of dsαGlu injected into insect's hemocoel than with 2 µg, and was clearly evident on the 4^th^, but not the 2^nd^ day after a blood meal. On day 4, the α-glucosidase activity is normally higher in relation to the day 2, when the PMM are also more abundant [22]. After checking the silencing efficiency by qPCR, we investigated whether dsαGlu injection would confer a readily detectable effect on enzyme activities in the *R. prolixus* midgut. Injection of dsαGlu decreased the α-glucosidase activity in the gut lumen of *R. prolixus* (Figure 3B). This effect was more evident on day 4 after a blood meal when insects were injected with 10 µg of dsαGlu before feeding. On day 2 after a blood meal, the decrease in Hz formation activity was not significantly different when insects were injected with 2 or 10 µg of dsαGlu. The decreasing of α-glucosidase gene expression and activity were accomplished by inhibition of Hz formation (Figure 3C). This inhibition was observed with 10 µg of dsαGlu, but not with 2 µg of dsαGlu. These results demonstrate that the knock-down effects were more evident on day 4 after feeding, when both activities of this enzyme were clearly compromised. These results clearly evidence that Hz formation is dependent upon the α-glucosidase enzyme, since knocking-down this enzyme similarly reduced both activities.

For experiment 2, double-stranded β-galactosidase *Escherichia coli* (dsLacZ), instead dsαGlu, was injected using the same procedure. Results showed that α-glucosidase gene expression, as revealed by qPCR analysis (Figure 3D), was not reduced. Analysis of α-glucosidase activity and Hz content demonstrated that injections of dsLacZ did not cause significant reductions in these parameters in comparison to control insects (Figures 3E and 3F).

**Figure 4:**
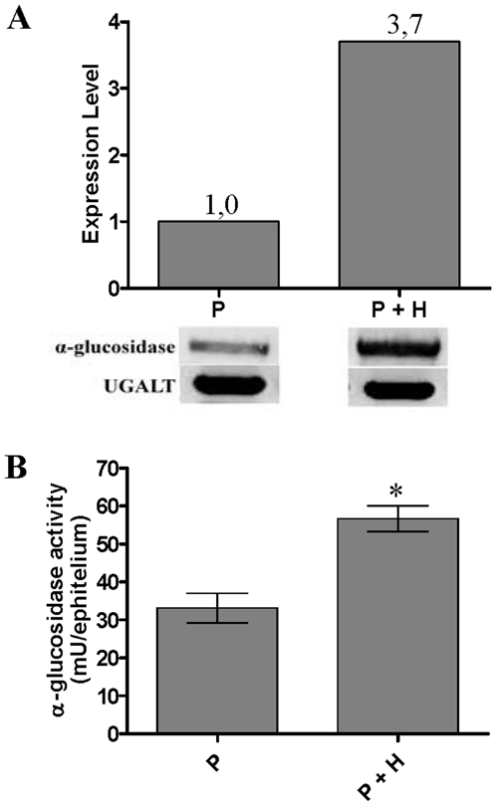
Influence of heme from meal on the α-glucosidase expression (A) and activity (B) in the *R. prolixus* midgut. P – control insects fed on plasma; P + H – insects fed on hemin-enriched plasma (500 μM). Insects were fed on rabbit plasma with and without hemin. Four days after feeding, midguts (n = 20) were dissected in cold 100 mM NaCl. The α-glucosidase activity was determined by measuring the release of ρ-nitrophenolate from ρ-nitrophenyl α-D-glucopyranoside. Results shown are representative of three independent experiments run in triplicate. Plasma plus hemin is significantly different from plasma *(*P*<0.05).

In order to further evaluation of the influence of heme on α-glucosidase activity, insects were fed either on plasma or plasma containing 500 µM hemin. The analysis of the pattern of α-glucosidase gene expression in the midgut of insects that fed on hemin-enriched plasma shows that hemin can induce α-glucosidase gene expression (Figure 4A). α-Glucosidase activity was also checked in both situations above. It was clear that α-glucosidase activity was higher in the midgut of insects that fed on hemin-enriched plasma (Figure 4B). These results suggest that heme may additionally play a role in gene expression of this enzyme.

### Cloning and sequencing of the α-glucosidase cDNA

**Figure 5:**
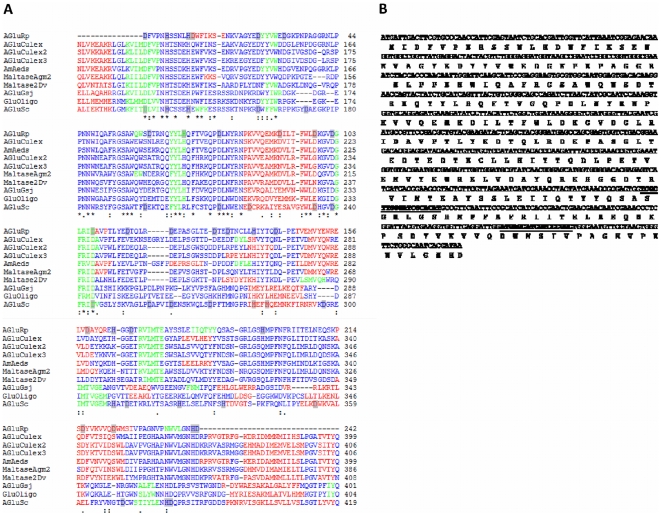
Analysis of the α-glucosidase sequence. A. Alignment of amino acid sequences of α-glucosidase from *R. prolixus* (AgluRp), *Culex quinquefasciatus* (AGluCulex, AGluCulex2, AGluCulex3), α-amylase from *Aedes aegypti* (AmAeds), maltase-like Agm2 from *Anopheles gambiae* (MaltaseAgm2), maltase 2 from *Drosophila virilis* (Maltase2Dv), α-Glucosidase from Gsj (AgluGsj), Oligo-1,6-Glucosidase from *Bacillus cereus* (GluOligo) and α-glucosidase from *Saccharomyces cerevisiae* (AgluSc). Identical residues are indicated by “*”; conserved and semiconserved residues are indicated by “:” and “.”, respectively. Residues of aspartic acid and histidine present in AGluRp and also present in AGluSc are marked in gray. The secondary structure prediction using the JPred server is represented in red (α-helices), green (β-sheets) and blue (loops). B. Partial nucleotide sequence of the *R. prolixus* α-glucosidase cDNA and its deduced amino acid sequence. The amino acid sequences used for the design of specific Real Time-PCR primers are underlined.

In order to characterize the enzyme structure, we cloned and sequenced the α-glucosidase cDNA. Figure 5B shows the nucleotide sequence and deduced *R. prolixus* α-glucosidase aminoacid sequence (GenBank FJ236283). The alignment with deduced α-glucosidases amino acid sequence from *Culex pipiens, Aedes aegypti, Drosophila melanogaster, Anopheles gambie, Geobacillus sp., Bacillus cereus* and *Saccharomyces cerevisae* showed that *R. prolixus* α-glucosidase presented 70% identity to *Culex*, 69% identity to *Aedes*, 59% to *Anopheles* and 24% identity to *Saccharomyces* (Table 1). The differences and similarities between insects, bacteria and yeast showed that *R. prolixus* α-glucosidase is more closely related to insect than to microbial enzyme. In this context, *R. prolixus* α-glucosidase is more closely related to α-glucosidases from other hematophagous insects such as *Culex* and *Aedes*.

**Table 1:** Physiological effects of dsRNA-mediated silencing of α-glucosidase.

Accession^a^	Putative Function b	E-value	Organism Similarity	Identities (%)
FJ236283	Alpha-glucosidase	3e-145	*Rhodnius prolixus*	100
XP_001851486	Alpha-glucosidase	8e-102	*Culex quinquefasciatus*	70
XP_001869889	Alpha-amylase	4e-93	*Culex quinquefasciatus*	64
XP_001847532	Alpha-glucosidase	5e-90	*Culex quinquefasciatus*	64
XP_001656785	Alpha-amylase	7e-101	*Aedes aegypti*	69
CAA60858	Maltose like protein Agm2	4e-86	*Anopheles gambie*	59
AAB82328	Maltase 2	1e-71	*Drosophila virilis*	52
PDB^c^ ZEO_A	Alpha-glucosidase	6e-47	*Geobacillus sp.*	27
PDB 1UOK_A	Oligo-1,6-Glucosidase	1e-25	*Bacillus cereus*	27
CAA87020	Alpha-glucosidase	2e-22	*Saccharomyces cerevisiae*	24

The insects were injected with 2 µL of 100 mM PBS pH 7.4 or dsLacZ (controls) and dsα-glu (2 or 10 µg/female); mortality and oviposition were monitored 4 days after feeding. In all panels, results are means ±SEM (n = 70). The insects injected with 10 µg dsα-glu, analyzed 4 days feeding, were significantly different from control insects injected with both PBS and dsLacZ and also analyzed 4 days after feeding ^*^(*P*<0.05).

a Access number to GenBank database.

b Function expected according the Blastx sequence results similarity in GenBank database.

c Protein Data Bank.

Previous studies indicated that *Plasmodium falciparum* Histidine Rich Protein-2 (PfHRP2) has an extraordinary capacity for binding heme [27], and it has been implicated in Hz formation [28]. It is noteworthy that the active site of α-glucosidases presents highly conserved aspartic and histidine residues [29]. Herein the analysis of *Rhodnius prolixus* α-glucosidase (AGluRp) shows conserved histidine and aspartic acid residues in the substrate-binding site of enzyme. However, a significant deviation in position of these key residues was observed when comparing *R. prolixus* with *S. cerevisae* (AGluSc) sequences (Figure 5A). By multiple alignment of α-glucosidase from insects, bacteria and yeast, we found that His69, Asp132 and Asp159 were not conserved in AGluSc suggesting the role of these residues in the process, since α-glucosidase from yeast did not present Hz formation activity (data not shown).

## Discussion

Hz formation, in addition to other strategies to overcome heme toxicity [5], is a very efficient mechanism for reducing heme availability in the midgut of hematophagous organisms.

Despite all the recent attempts to characterize Hz formation in *Plasmodium* parasites and other models, this is still a controversial issue, as many factors have been suggested to promote Hz synthesis. The consequence of these many views on the Hz formation is that there has been no consensus on the mechanism of Hz formation *in vivo* [30]. Here, we show that α-glucosidase promotes Hz formation in the midgut of *R. prolixus*. The presence of an enzyme was implied earlier in the process, since a crude extract from trophozoites promoted Hz formation *in vitro*, being apparently sensitive to heat, and disappearing if the sample was boiled [31]. Recently, a new HDP was described in *Plasmodium* [19]; this protein may be an additional element for speeding up the process of Hz formation in this model, since the protein moiety would play a role in the nucleation. These recent data show that the role of proteins can not be ruled out, although the process of Hz formation may be observed from another point of view in different models.

The first evidence that prompted us to suggest the involvement of an α-glucosidase in the process of Hz formation was the strong correlation observed between the enzyme activity and Hz content in the midgut of *R. prolixus*, following the days after a blood meal [22]. *In vivo*, PMM production is accompanied by increments in Hz content. This activity was associated mainly to a protein fraction of midgut. It is reasonable, therefore, to conclude that a protein extract has the active component that promotes the Hz formation. It is probable that the hydrophobic environment of the membrane may favor its activity, since when both components (lipids and proteins) were present together in the Hz formation assay, a synergistic effect was observed [22]. Assays of the enzyme in the presence of specific α-glucosidase inhibitors confirmed the direct relationship between a carbohydrase and Hz formation activities. DEPC inhibition suggests the involvement of histidine residues in both activities, although Lynn [32] has suggested that aspartic acid is more important for the pH required for Hz formation. According to our data, and to the physicochemical requirements to Hz formation, proteins would play a role as a nucleation site for crystal growth [30]. This is a feasible hypothesis since the presence of α-glucosidase inhibitors, after the assay has been started, did not avoid Hz formation activity. In addition, boiling of the assay mixture 10 hours after the assay taking place had also no inhibitory effect on the growing of Hz content. Confirming the inhibitory effects of both boiling and presence of α-glucosidase inhibitors at the beginning of Hz formation assay, we can conclude that this enzyme acts as a nucleation site, initiating the process of Hz formation (Figure 1). Once started, Hz formation can goes on even in the absence of it. Since sufficient nucleation has taken place, growth of β-hematin begins to dominate and rapid conversion occurs [30].

Maltose is specifically cleaved to glucose by α-glucosidase [33]. Analyzing the role of maltose in the Hz formation activity, to support or not, the hypothesis that heme could bind to the same α-glucosidase substrate-binding site, competition assays were performed. The Hz formation was largely decreased when maltose was pre-incubated with α-glucosidase 4 hours before adding hemin. In such case the maltose appears to behave like a tight-binding reversible competitive inhibitor, preventing access of hemin to the substrate-binding site, since the tested maltose concentration was sufficiently higher than that of hemin (Figure 2).

Silencing of the α-glucosidase gene by injection of dsαGlu resulted in a reduction of both α-glucosidase activity and Hz formation in a very similar pattern. The phenotypes were analyzed 2 and 4 days after a blood meal. However, the RNA interference (RNAi) effect was only evident at 4 days after a blood meal. It is known that α-glucosidase activity is low at initial stages of blood digestion in the *R. prolixus* midgut [22]. Similarly, Hz formed in the gut lumen is also low at this period. At day 2 after feeding, it is probable that the basal level of α-glucosidase mRNA is low, corresponding to a very discrete RNAi effect. However, on day 4 after feeding, the level of α-glucosidase mRNA is higher, as reflected by α-glucosidase activity, resulting in more evident silencing (Figures 3A and 3B). Concomitant with a reduction in α-glucosidase activity, it was also detected a reduction in the Hz formed in the *R. prolixus* midgut at 4 days after feeding, demonstrating that the knocked-down enzyme compromised the ability to form Hz in the insect midgut (Figure 3C). Interestingly, during our RNAi experiments 44% dead insects were commonly observed at 4 days after feeding (Table 2), suggesting a strong action of α-glucosidase silencing in vital physiological process. These results are in agreement with the α-glucosidase gene expression, which was reduced in the injected insects. To our knowledge, this is the first demonstration of a carbohydrase involved in heme detoxification. These results provide a physiological significance to this enzyme in hematophagous insects, for instance. It was also visible the strong reddish aspect of hemolymph of prostrated insects which had been injected with dsα-Glu (data not shown). The reddish aspect may reflect the effect of heme passage from midgut to hemocoel due to high free heme concentration that is not sequestered into Hz. By inhibiting Hz formation in the *R. prolixus* midgut, higher levels of free heme could get into the hemocoel increasing its pro-oxidant effects which lead to increased risk of physiological damages [6].

**Table 2:** Analysis of α-glucosidase genes using Blastx.

	PBS 4 days	dsLacZ 10 µg	dsαGlu 2 µg	dsαGlu 10 µg
**Mortality (%)**	10	20	25	44^*^
**Oviposition (eggs/female)**	25	22	16	10^*^

Glycosidases are the primary enzymes for digestion in the plant-feeding insect, *S. gregaria* [34], but the presence of comparatively high activities in the hematophagous insects, such as *Phlebotomus papatasi* [35] and *Aedes aegypti* [36], suggests that glycosidases are also important for hematophagous insects, despite their low carbohydrate diet. In *R. prolixus*, hemin caused increases in both α-glucosidase activity and gene expression, suggesting that heme may be an important regulator for α-glucosidase synthesis (Figures 4A and 4B); this could be of high value as an adaptative mechanism to deal with a high content of heme during blood digestion. Dillon and Kordy [37] compared the α-glucosidase activities from the midgut of blood and sucrose-fed sandflies. Their results revealed a much higher specific activity in the midgut of blood-fed insects. It is probable that this enzyme plays a role in the heme detoxification in this model as well, and deserves attention.

Sequencing of the *R. prolixus* α-glucosidase cDNA clone showed that its deduced amino acid sequence presents a high identity with α-glucosidases from other hematophagous insects (Figure 5A and Table 2), reinforcing the idea of its importance in different models. Another important finding is the conserved amino acid residues at active sites of insect α-glucosidase sequences, such as histidine, aspartic and glutamic acid. However, some of these key residues were not observed in yeast enzyme. This structural difference may be related to changes in the active site geometry and substrate specificity as well as the catalytic efficiency. The high proportion of aspartic acid is suggestive of a role for this amino acid in the heme binding process at pHs lower than 6.0 [32]. At first glance, this suggests that the site of the enzyme which accommodates the heme might contain a carboxylate residue. Metal binding properties are associated with amino acids containing carboxylate, imidazole and hydroxyl side chains [38]. In *R. prolixus* the heme-binding to the enzyme might involve both histidine and aspartic acid residues in the substrate-binding site.

Although much evidence suggests lipids to be important catalysts in the Hz formation, since it is clearly produced first as a crystalline dimer, Egan reports that the role of proteins should be viewed in a new light [30]. Since a crystalline structure growth is indeed an autocatalytic process, nucleated Hz can grow in a lipid environment. However, it is yet uncertain how nucleation occurs; it may be that it is mediated by proteins, explaining their role in this process [30]. As such, it is reasonable to suppose that α-glucosidase plays a role as a heme nuclease for Hz formation in *R. prolixus* midgut. Since this is a PMM-bound enzyme, it is possible that α-glucosidase starts to bind heme and nucleate Hz (Figures 1C and 1D), just after heme release from hemoglobin digestion, at the interface between membrane and the aqueous phase in the gut lumen, as demonstrated and discussed as an important requirement for Hz formation [30], [39]. This theory is in accordance with transmission electron micrographies viewed by Oliveira *et al.* [14], who described Hz formed within vesicles bounded by a bilayer membrane. Reinforcing this interpretation, Silva *et al.* [20] showed that a polyclonal antibody raised against *D. peruvianus* α-glucosidase [40] also immunolocalized an α-glucosidase on *R. prolixus* PMM. We herein demonstrate that this same antibody inhibits Hz formation by *R. prolixus* α-glucosidase (Figure 1). The ability to catalyze transglycosylation reactions is a hallmark of α-glucosidases [41], thus, it may form an iron-carboxylate bound that is required for Hz formation.

In the course of evolution, organisms that have selected blood as their main source of nutrients, consequently selected an array of strategies to overcome heme toxicity and adapt them to this alimentary habit [5]. It is known that hematophagy has appeared independently several times during the evolution of arthropods [42], where different groups of present-day hematophagous organisms are derived from non-hematophagous ancestors. For the success of these organisms, some pre-existent adaptations were necessary, such as the development of mouthparts capable of piercing and cutting [43]. It is important to recognize that some physiological features have been indicated as playing an important role in successfully feeding on blood [44]. In this case, if we imagine that some hematophagous insects are derived from phytophagous insects, pre-adapted to suck sap, the conservation of enzymes such as α-glucosidases would represent one of the more important pre-conditions in conferring fitness to hematophagy. Our findings corroborate to this idea, showing that the conserved α-glucosidase primary structure may have been an additional important pre-condition for some groups of hematophagous insects with acidic digestion.

To understand Hz formation in the gut lumen of *R. prolixus*, the present study provided important data to complement the points discussed in the review by Egan [30]. In this context, α-glucosidase may start to bind heme just during its release from hemoglobin, during its digestion, avoiding heme precipitation at the acidic pH in the *R. prolixus* midgut. It should be remembered that proteolytic enzymes such as cathepsin B-like proteinase, cathepsin D-like proteinase and carboxypeptidase A and B are secreted to maximal activity at 6–7 days after feeding [45], acting on hemoglobin in the luminal space. During this same period, α-glucosidase presents an elevated activity with an elevated production of PMM as well [22], [46]. Increasing α-glucosidase activity during blood digestion seems to be a recurrent feature among hematophagous insects [35], [37]. Taken together, it is feasible that the release of heme from hemoglobin is rapidly coupled to nucleation by the α-glucosidase at the PMM, where the process of Hz crystal growth is sustained in a low dielectric medium. The investigation of this activity played by α-glucosidase could lead to an additional target for new drug design and vector control strategies.

## Materials and Methods

### Animals

*R. prolixus* was kept at 28°C and 80% relative humidity. The *Rhodnius* females were fed every 21 days with rabbit blood or plasma, using an artificial feeder described by Garcia [47]. Insects fed on plasma were first fed twice on blood in order to guarantee that they were physiologically mature. For experiments, only adult female insects were used.

### Protein Extraction

Midguts from 150 insects at 4 days after a plasma meal were obtained and extensively washed 0.15M NaCl. Insect midguts were homogenized in distilled water using a Potter-Elvehjem glass homogeneizer, followed for repeated sessions of freezing and unfreezing of the samples in liquid nitrogen. The samples were centrifuged at 18,000 x*g* for 30 min at 4°C at each freezing cycle. Solubilization of membrane-bound proteins was carried out by treatment with 0.1% Nonidet P-40 (NP-40) for 12 h at 4°C in the 20 mM sodium phosphate buffer pH 7.4, 0.1%, 5 mM imidazole, 1 mM PMSF and 1 mM benzamidine. The sample was incubated overnight in the same buffer under agitation at 4°C. After centrifuging at 18,000 *g* for 30 min at 4°C, protein concentrations were determined by BCA [48].

### Hemozoin formation assay

Midgut epithelium and contents from rabbit blood and plasma fed *R. prolixus* were obtained 2 or 4 days after feeding in cold 0.15 M NaCl and centrifuged at 20,000 xg for 20 min at 4°C. The supernatant was discarded and sediment homogenized and washed in cold 0.15 M NaCl three times. Later this material was centrifuged at 20,000 xg for 20 min at 4°C. Samples corresponding to 15 µg/mL of protein (protein extract) were incubated for 24 h at 28°C in 0.5 M sodium acetate, pH 4.8, in the presence of 100 µM hemin. After incubation, the reaction mixture was centrifuged at 15,000 xg for 15 min at 25°C. The sediments were washed three times with 1 mL f 0.1 M NaHCO_3_ +2.5% SDS, pH 9.1, and twice with deionized water. The final sediments were solubilized in 0.1 M NaOH and the amount of heme determined spectrophotometrically at 400 nm in a GBC-UV/Vis-920 spectrophotometer.

### Hemozoin extraction

Hz was extracted from the midgut of *R. prolixus*, as previously described [2]. Midgut contents from 6 insects at 2 and 4 days after a blood or plasma meal were obtained by gently washing the dissected midguts in 0.15 M NaCl. Tissue was discarded and the suspensions were centrifuged at 20,000 g for 20 min. The insoluble pigment was further purified by three washes with 0.1 M NaHCO_3_ +2.5% SDS, pH 9.1. Final sediments were solubilized in 0.1 M NaOH and the amount of heme determined spectrophotometrically at 400 nm in a GBCUV/Vis-920 spectrophotometer.

### Assay of α-glucosidase activity

α-Glucosidase activity was determined using p-nitrophenyl α-D-glucopyranoside (10 mM) (Sigma Ltd.) in 100 mM citrate phosphate buffer pH 5.5 as substrate and by following the appearance of p-nitrophenolate, according to the method of Terra [49]. All assays were performed at 30°C. Incubations were carried out for at least four different periods of time (15, 30, 45 and 60 min). Reactions were stopped with 200 µL of 0.5 M Na_2_CO_3_ and initial rates of hydrolysis were calculated. The absorbance of released *p*-nitrophenolate was read in a GBC-UV/Vis-920 spectrophotometer at 405 nm. One unit of enzyme was defined as the amount required to hydrolyze 1 µmol of substrate per minute in the assay conditions.

### Ion exchange chromatography for purification of α-glucosidase

Protein extract of midgut epithelium from *R. prolixus* fed on rabbit blood was subjected to ion exchange chromatography in a Mono Q HR 5/5 column installed in a Perkin–Elmer FPLC equipment, and equilibrated with HEPES buffer, pH 7.2, containing 0.1% NP-40. The column was washed with the same buffer and the enzyme was eluted with a linear gradient of 0–0.5 M NaCl in HEPES buffer at a flow rate of 0.5 mL/min [50]. One-milliliter fractions were collected and used for measurements of enzyme activity by a colorimetric method (see below).

### Construction of double-stranded RNA for RNAi

The MEGAscript® RNAi Kit (Ambion) was used to generate double-stranded RNA α-glucosidase (dsαGlu), according to the manufacturer's instructions. For synthesis of dsαGlu, degenerated primers for the *Anopheles aquasalis* α-glucosidase gene Aglu F (5′ ATA/C/T T/CTN GAC/T TTT/C GTN CCN AAC/T CAC/T 3′) and Aglu R (5′ G/ATC G/ATG G/ATT NCC NAA/G NAC CCA G/ATT 3′) were used as templates. Each reaction contained 1 µg of each plasmid with specific sense or antisense inserts, nucleotides (ATP, CTP, GTP, UTP) and T7 polymerase enzyme, and was performed and incubated for 16 h at 37°C. These samples were incubated at 75°C for 5 min and cooled at room temperature. After double-stranded RNA (dsRNA) formation, the material was treated by DNAse/RNAse, at 37°C for 1 h. The dsRNA was recouped in 10 mM Tris-HCl pH 7, 1 mM EDTA buffer that was compatible with injection. The resulting dsRNA was analyzed by 1% agarose gel electrophoresis and concentrations were determined by spectrophotometry (280 nm).

### Alpha-glucosidase silencing using double-stranded RNA

Two µL of dsαGlu at different dilutions (2 or 10 µg) were intrathoracically injected into adult females' hemocoel using a Hamilton syringe. The insects used in the experiments were first fed twice on rabbit blood and injected 21 days after last feeding. Insects were fed on rabbit blood 2 h after injection. After 4 days at 28°C and 80% humidity, midguts were dissected in their own hemolymph and luminal contents were collected in cold 0.1 M sodium phosphate buffer (PBS at 4°C), pH 7.4. Control insects were injected with PBS or dsRNA β-galactosidase *Escherichia coli* (dsLacZ) [51] obtained as described for dsαGlu.

### RNA extractions and RT-PCR

Total RNA was extracted from *R. prolixus* midgut using TRIZOL (Invitrogen, Carlsbad, CA). Synthesis of cDNA was performed using the reverse transcriptase Superscript III system (Invitrogen), according to the manufacturer instructions. Real Time-PCR was carried out using specific primers, Aglu F (5′TCGCTTGGGATCGCACAT 3′) and Aglu R (5′GCCGGGACGATGCTCAT 3′), to amplify a fragment of 114 bp of the Aglu transcript and the primers 18S F (5′GTT GGTATTGATGTACGCTGGA 3′) and 18S R (5′CCTACGGAAACCTTGTTACGA 3′) to amplify the 18S RNAr transcript, used as an internal control [36]. To verify α-glucosidase expression in the presence or absence of heme, RT-PCR was carried out using degenerated primers for the *Anopheles aquasalis* α-glucosidase gene and UDP-galactose Transporter UGALT F (5′ATGGCACTCCAGTGGGTTAG 3′) and UGALT R (5′AAGAAAGGCGAGGCATTGTA 3′) as housekeeping [52]. Ratiometric densitometry data were obtained through the TotalLab TL100 software from Nonlinear Dynamics.

### Relative gene expression as determined by Real-time PCR analysis

Real-time PCR was carried out using cDNA pools of six female adults (six midguts) from each group. The samples were assayed with the LightCycler 2.0 instrument (Roche Diagnostics). Reactions were carried out in a total volume of 10 µL with a final concentration of 5 pmol primer. Relative expressions of gene were assayed with the Multiple Condition Solver REST-MSC© - version 2, based on the standard curve. The standard curves were generated using five serial 2-fold dilutions of the sample that reached exponential amplification at the earlier cycle. The number of copies of the analyzed gene were expressed relative to the **R = (E_target_)^ΔCPtarget^** ^***(MEAN control – MEAN sample)***^ ***/*** **(E_ref_)** **^ΔCPref^** ^***(MEAN control – MEAN sample)***^. The Real-time PCR products were analyzed by 1.3% agarose gel electrophoresis.

### Cloning and sequencing of α-glucosidase cDNA

For PCR amplification of a segment of the gene encoding α-glucosidase degenerated primers for the *Anopheles aquasalis* α-glucosidase gene were using. The amplified 0.75-kb DNA fragment was cloned into the ddT site of the plasmid pTZ57R/T and sequenced using the InsT/Aclone™ PCR Product Cloning Kit (Fermentas Life Sciences), according to the manufacturer's instructions.

### Statistical analysis

Comparisons between groups were made by the non-paired Student's t test, using GraphPad Prism. For all tests, a difference of P<0.05 was considered to be significant.

## References

[1] Oliveira PL, Oliveira MF (2002). Vampires, Pasteur and reactive oxygen species. Is the switch from aerobic to anaerobic metabolism a preventive antioxidant defence in blood-feeding parasites?. FEBS Letters.

[2] Oliveira MF, Silva JR, Dansa-Petretski M, de Souza W, Lins U (1999). Haem detoxification by an insect.. Nature.

[3] Lara FA, Lins U, Paiva-Silva G, Almeida IC, Braga CM (2003). A new intracellular pathway of haem detoxification in the midgut of the cattle tick *Boophilus microplus*: aggregation inside a specialized organelle, the hemosome.. J Exp Biol.

[4] Pascoa V, Oliveira PL, Dansa-Petretski M, Silva JR, Alvarenga PH (2002). *Aedes aegypti* peritrophic matrix and its interaction with heme during blood digestion.. Insect Biochem Mol Biol.

[5] Graça-Souza AV, Maya-Monteiro C, Paiva-Silva GO, Braz GR, Paes MC (2006). Adaptations against heme toxicity in blood-feeding arthropods.. Insect Biochem Mol Biol.

[6] Oliveira MF, Silva JR, Dansa-Petretski M, de Souza W, Braga CM (2000a). Haemozoin formation in the midgut of the blood-sucking insect *Rhodnius prolixus*.. FEBS Letters.

[7] Francis SE, Sullivan  DJ, Goldberg DE (1997). Hemoglobin metabolism in the malaria parasite *Plasmodium falciparum*.. Annu Rev Microbiol.

[8] Oliveira MF, d'Avila JC, Torres CR, Oliveira PL, Tempone AJ (2000b). Haemozoin in *Schistosoma mansoni*.. Mol Biochem Parasitol.

[9] Pisciotta JM, Ponder EL, Fried B, Sullivan D (2005). Hemozoin formation in *Echinostoma trivolvis* rediae.. Int J Parasitol.

[10] Chen MM, Shi L, Sullivan JRDJ (2001). *Haemoproteus* and *Schistosoma* synthesize heme polymers similar to *Plasmodium* hemozoin and beta-hematin.. Mol Biochem Parasitol.

[11] Fitch CD, Cai GZ, Chen YF, Shoemaker JD (1999). Involvement of lipids in ferriprotoporphyrin IX polymerization in malaria.. Biochim Biophys Acta.

[12] Jackson KE, Klonis N, Ferguson DJ, Adisa A, Dogovski C (2004). Food vacuole-associated lipid bodies and heterogeneous lipid environments in the malaria parasite, *Plasmodium falciparum*.. Mol Microbiol.

[13] Pisciotta JM, Coppens I, Tripathi AK, Scholl PF, Shuman J (2007). The role of neutral lipid nanospheres in *Plasmodium falciparum* haem crystallization.. Biochem J.

[14] Oliveira MF, Kycia SW, Gomez A, Kosar AJ, Bohle DS (2005). Structural and morphological characterization of hemozoin produced by *Schistosoma mansoni* and *Rhodnius prolixus*.. FEBS Lett.

[15] Egan TJ, Chen JY, de Villiers KA, Mabotha TE, Naidoo KJ (2006). Haemozoin (beta-haematin) biomineralization occurs by self-assembly near the lipid/water interface.. FEBS Lett.

[16] Sullivan  DJ, Gluzman IY, Goldberg DE (1996). Haemozoin formation mediated by histidine-rich proteins.. Science.

[17] Pandey AV, Babbarwal VK, Okoyeh JN, Joshi RM, Puri SK (2003). Hemozoin formation in malaria: a two-step process involving histidine-rich proteins and lipids.. Biochem Biophys Res Commun.

[18] Sullivan DJ (2002). Theories on malarial pigment formation and quinoline action.. Int J Parasitol.

[19] Jani D, Nagarkatti R, Beatty W, Angel R, Slebodnick C (2008). HDP - a novel heme detoxification protein from the malaria parasite.. PLoS Pathog.

[20] Silva CP, Silva JR, Vasconcelos FF, Petretski MD, Damatta RA (2004). Occurrence of midgut PMM in paraneopteran insect orders with comments on their function and evolutionary significance.. Arthropod Struc Dev.

[21] Okuyama M, Okuno A, Shimizu N, Mori H, Kimura A (2001). Carboxyl group of residue Asp647 as possible proton donor in catalytic reaction of alpha-glucosidase from *Schizosaccharomyces pombe*.. Eur J Biochem.

[22] Silva JR, Mury FB, Oliveira MF, Oliveira PL, Silva CP (2007). Perimicrovillar membranes promote hemozoin formation into *Rhodnius prolixus* midgut.. Insect Biochem Molec Biol.

[23] Silva CP, Terra WR (1994). Digestive and absorptive sites along the midgut of the cotton seed sucker bug *Dysdercus peruvianus*.. Insect Biochem Mol Biol.

[24] Romaniouk A, Vijay IK (1997). Structure-function relationships in glucosidase I: amino acids involved in binding the substrate to the enzyme.. Glycobiology.

[25] Zeng YC, Elbein AD (1998). Purification to homogeneity and properties of plant glucosidase I.. Arch Biochem Biophys.

[26] Dhanawansa R, Faridmoayer A, Van Der Merwe G, Li YX, Scaman CH (2002). Overexpression, purification, and partial characterization of *Saccharomyces cerevisiae* processing alpha-glucosidase I.. Glycobiology.

[27] Choi CYH, Cerda JF, Chu H-A, Babcock GT, Marletta MA (1999). Spectroscopic characterization of the heme-binding sites in *Plasmodium falciparum* histidine-rich protein 2.. Biochemistry.

[28] Schneider EL, Marletta MA (2005). Heme Binding to the Histidine-Rich Protein II from *Plasmodium falciparum*.. Biochemistry.

[29] Shirai T, Hung VS, Morinaka K, Kobayashi T, Ito S (2008). Crystal structure of GH13 α-glucosidase GSJ from one of the deepest sea bacteria.. Proteins.

[30] Egan TJ (2008). Recent advances in understanding the mechanism of hemozoin (malaria pigment) formation.. J Inorg Biochem.

[31] Slater AF, Cerami A (1992). Inhibition by choroquine of a novel haem polymerase enzyme e activity in malaria trophozoites.. Nature.

[32] Lynn A, Chandra S, Malhotra P, Chauhan VS (1999). Heme binding and polymerization by *Plasmodium falciparum* histidine rich protein II: influence of pH on activity and conformation.. FEBS Lett.

[33] Dixon M, Webb EC (1979). Enzymes..

[34] Morgan MRJ (1975). A qualitative survey of the carbohydrases of the alimentary tract of the migratory locust, *Locusta migratoria migratorioides*.. J Insect Physiol.

[35] Jacobson RL, Schlein Y (2001). *Phlebotomus papatasi* and *Leishmania major* parasites express alpha-amylase and alpha-glucosidase.. Acta Trop.

[36] Whitby K, Pierson TC, Geiss B, Lane K, Engle M (2005). Castanospermine, a potent inhibitor of dengue virus infection *in vitro* and *in vivo*.. J Virol.

[37] Dillon RJ, el-Kordy E (1997). Carbohydrate digestion in sandflies: alpha-glucosidase activity in the midgut of *Phlebotomus langeroni*.. Comp Biochem Physiol B Biochem Mol Biol.

[38] Tainer JA, Roberts VA, Getzo ED (1992). Protein metal-binding sites.. Curr Opin Biotechnol.

[39] Pisciotta JM, Sullivan D (2008). Hemozoin: oil versus water.. Parasitol Int.

[40] Silva CP, Ribeiro AF, Gulbenkian S, Terra WR (1995). Organization, origin and function of the outer microvillar (perimicrovillar) membranes of *Dysdercus peruvianus* (Hemiptera) midgut cells.. J Insect Physiol.

[41] Ferrer M, Golyshina OV, Plou FJ, Timmis KN, Golyshin PN (2005). A novel alpha-glucosidase from the acidophilic archaeon *Ferroplasma acidiphilum* strain Y with high transglycosylation activity and an unusual catalytic nucleophile.. Biochem J.

[42] Ribeiro JM (1995). Blood-feeding arthropods: live syringes or invertebrate pharmacologists?. Infect Agents Dis.

[43] Lehane MJ, Lehane MJ (2005). The evolution of the blood-sucking habit.. Biology of Blood-Sucking Insects.

[44] Ribeiro JM, Francischetti IM (2003). Role of arthropod saliva in blood feeding: sialome and post-sialome perspectives.. Annu Rev Entomol.

[45] Terra WR (1988). Physiology and biochemistry of insect digestion: an evolutionary perspective.. Braz J Med Biol Res.

[46] Billingsley PF, Downe AER (1983). Ultrastutural changes in posterior midgut cells associated with blood feeding in adult female *Rhodnius prolixus* Stal (Hemiptera: Reduviidae).. Can J Zool.

[47] Garcia ES, Macarini JD, Garcia MLM, Ubatuba FB (1975). Feeding of *Rhodnius prolixus* in the laboratory.. An Acad Bras Cienc.

[48] Smith PR, Krohn RI, Hermanson GT, Mallia AK, Gartner FH (1985). Measurements of protein using bicinchonic acid.. Anal Biochem.

[49] Terra WR, Ferreira C, Garcia ES (1979). Carbodiimide-reactive carboxyl groups at the active site of an insect midgut trehalase.. Biochim Biophys Acta.

[50] Bravo-Torres JC, Villagómez-Castro JC, Calvo-Méndez C, Flores-Carreón A, López-Romero E (2004). Purification and biochemical characterisation of a membrane-bound α-glucosidase from the parasite *Entamoeba histolytica*.. Int J Parasitol.

[51] Brandt SM, Jaramillo-Gutierrez G, Kumar S, Barillas-Mury C, Schneider DS (2008). Use of a *Drosophila* model to identify genes regulating *Plasmodium* growth in the mosquito.. Genetics.

[52] Ribeiro JM, Andersen J, Silva-Neto MA, Pham VM, Garfield MK (2004). Exploring the sialome of the blood-sucking bug *Rhodnius prolixus*.. Insect Biochem Mol Biol.

