# Correction: Alpha-Glucosidase Promotes Hemozoin Formation in a Blood-Sucking Bug: An Evolutionary History

**DOI:** 10.1371/annotation/f24ebd58-a620-4dad-a213-73a53ed6e1a0

**Published:** 2009-10-09

**Authors:** Flávia Borges Mury, José Roberto da Silva, Ligia Souza Ferreira, Beatriz dos Santos Ferreira, Gonçalo Apolinário de Souza-Filho, Jayme Augusto de Souza-Neto, Paulo Eduardo Martins Ribolla, Carlos Peres Silva, Viviane Veiga do Nascimento, Olga Lima Tavares Machado, Marília Amorim Berbert-Molina, Marilvia Dansa-Petretski

The titles and legend for Tables 1 and 2 were reversed.

The correct Table 2 can be viewed here: 

**Table 2:**
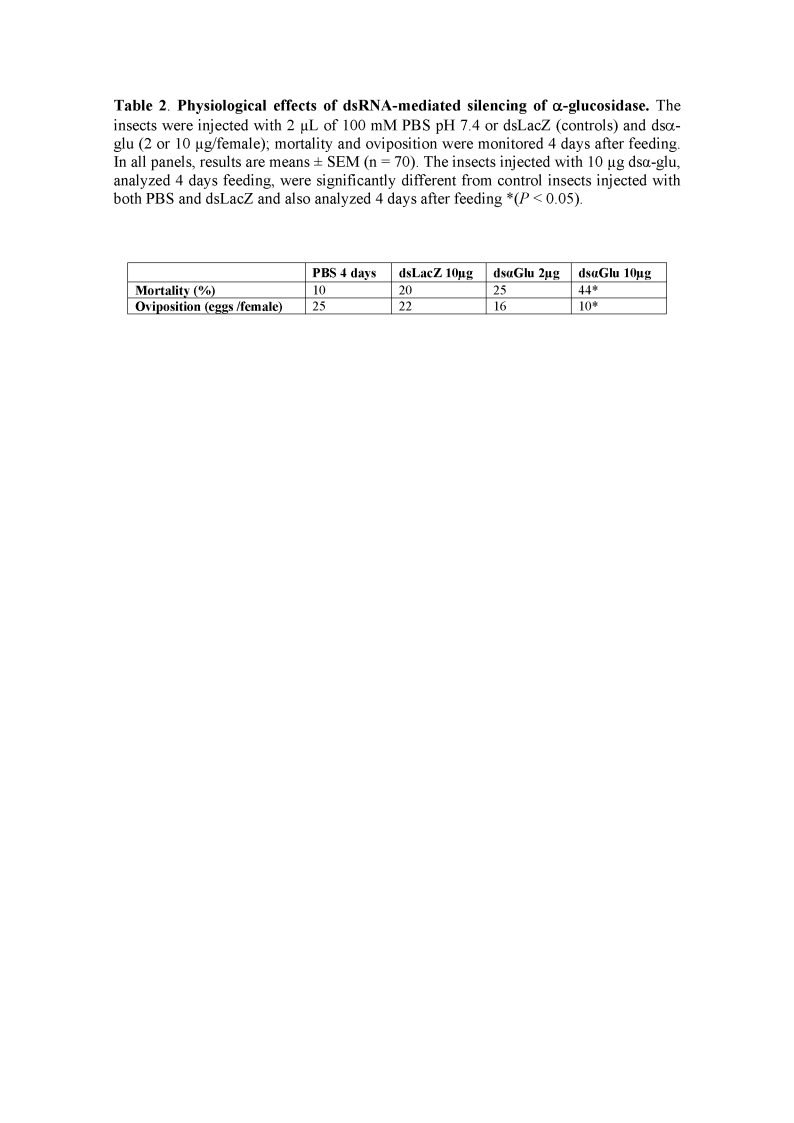
Physiological effects of dsRNA-mediated silencing of α-glucosidase. The insects were injected with 2 µL of 100 mM PBS pH 7.4 or dsLacZ (controls) and dsα-glu (2 or 10 µg/female); mortality and oviposition were monitored 4 days after feeding. In all panels, results are means +/-SEM (n = 70). The insects injected with 10 µg dsα-glu, analyzed 4 days feeding, were significantly different from control insects injected with both PBS and dsLacZ and also analyzed 4 days after feeding *(P<0.05).

