# Correction: Alpha-Glucosidase Promotes Hemozoin Formation in a Blood-Sucking Bug: An Evolutionary History

**DOI:** 10.1371/annotation/5a9be3cc-2441-4e00-bc5a-8653a14c0238

**Published:** 2009-10-09

**Authors:** Flávia Borges Mury, José Roberto da Silva, Ligia Souza Ferreira, Beatriz dos Santos Ferreira, Gonçalo Apolinário de Souza-Filho, Jayme Augusto de Souza-Neto, Paulo Eduardo Martins Ribolla, Carlos Peres Silva, Viviane Veiga do Nascimento, Olga Lima Tavares Machado, Marília Amorim Berbert-Molina, Marilvia Dansa-Petretski

The titles and legend for Tables 1 and 2 were reversed.

The correct Table 1 can be viewed here: 

**Table 1:**
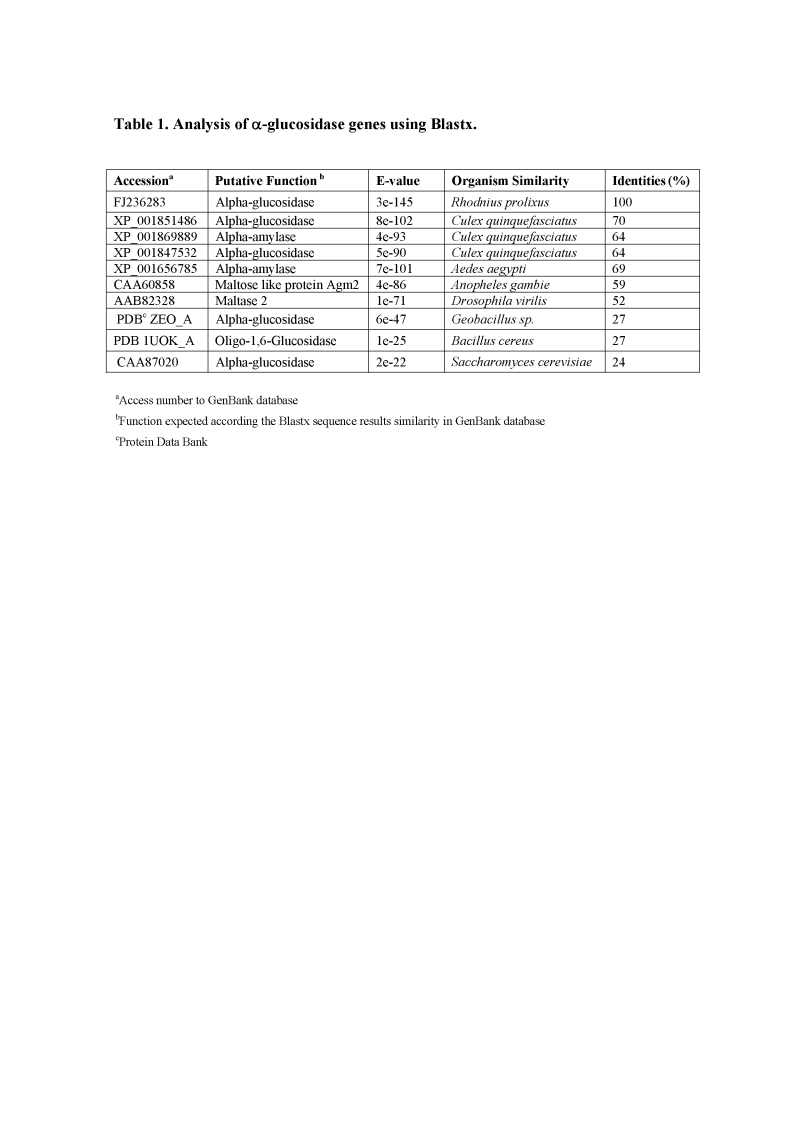
Analysis of alpha-glucosidase genes using Blastx.

